# Stromal fibroblasts in the microenvironment of gastric carcinomas promote tumor metastasis via upregulating TAGLN expression

**DOI:** 10.1186/1471-2121-14-17

**Published:** 2013-03-19

**Authors:** Beiqin Yu, Xuehua Chen, Jianfang Li, Ying Qu, Liping Su, Yibing Peng, Jian Huang, Jun Yan, Yingyan Yu, Qinlong Gu, Zhenggang Zhu, Bingya Liu

**Affiliations:** 1 Key Laboratory of Shanghai Gastric Neoplasms, Department of Surgery, Shanghai Institute of Digestive Surgery, Ruijin Hospital, Shanghai Jiao Tong University School of Medicine, Shanghai 200025, China; 2 Department of Laboratory Medicine, Ruijin Hospital, Shanghai Jiaotong University School of Medicine, Shanghai 200025, China; 3 National Human Genome Center at Shanghai, Shanghai 201203, China; 4 James Graham Brown Cancer Center, University of Louisville, Louisville, KY 40202, USA

**Keywords:** TAGLN, Fibroblast, Microenvironment, Tumor metastasis, Gastric carcinoma

## Abstract

**Background:**

Fibroblasts play a critical role in tumorigenesis, tumor progression and metastasis. However, their detailed molecular characteristics and clinical significance are still elusive. TAGLN is an actin-binding protein that plays an important role in tumorigenesis.

**Results:**

We investigated the interaction between cancer cells and the tumor microenvironment to determine how the fibroblasts from human gastric carcinoma facilitate tumorigenesis through TAGLN. QRT-PCR and Western blot indicated that TAGLN expression was upregulated in gastric carcinoma-associated fibroblasts (CAFs) that promote gastric cancer cell migration and invasion. Using small interfering RNA (siRNA), we found that CAFs enhanced tumor metastasis through upregulated TAGLN in vitro and in vivo. The expression of matrix metalloproteinase-2 (MMP-2) was significantly lower after TAGLN knock-down by siRNA. TAGLN levels were elevated in human gastric cancer stroma than normal gastric stroma and associated with differentiation and lymph node metastasis of gastric cancer.

**Conclusion:**

CAFs may promote gastric cancer cell migration and invasion via upregulating TAGLN and TAGLN induced MMP-2 production.

## Background

Gastric carcinoma is the second most common causes of cancer-related deaths in the world, especially in Asia [1,2]. Most of gastric cancer patients died of tumor recurrence caused by distant metastasis. Metastasis is a multi-stage process by which cancer cells disseminate from the primary neoplasm and invade surrounding tissue and distance organs, involving cancer cell motility, intravasation, transit in the blood or lymph and extravasation [3]. This process depends tightly on the surrounding microenvironment and tumor development relies on a continuous cross-talk between cancer cells and extracellular microenvironments [4].

Transgelin (TAGLN, also known as SM22) is an actin cross-linking protein that is involved in calcium interactions and regulates contractile properties [5]. It may play a role in cell differentiation, cell migration cell invasion and matrix remodeling by stabilizing the cytoskeleton through actin binding [6,7]. Overexpression of TAGLN protein has been observed in carcinomas of the stomach, liver, and esophagus, while decreased levels of TAGLN mRNA have been observed in breast and colon cancer cell lines and primary tumors [8].

It is generally accepted that tumor-stroma interactions play an important role in tumor development and progression. The stroma is constituted mainly of extracellular matrix (ECM) and cellular elements, such as fibroblasts [9,10]. The cellular components of the stroma are well-recognized as having a supportive role in carcinogenesis [11,12]. These stromal elements act in a synergistic cross-talk with cancer cells from the primary sites to sustain cancer growth and metastasis [13,14]. The tumor stroma which is referred to as a “reactive stroma” is associated with an increased number of fibroblasts, enhanced capillary density and deposition of a new ECM rich in type-1-collagen and fibrin [15]. Accumulating evidence reveals that stromal cells, such as fibroblasts have a more profound influence on the development and progression of carcinoma than was previously appreciated [15-17].

Fibroblasts are well-known to play roles in tumorigenesis and progression. Fibroblasts express vimentin, fibronectin, α-smooth-muscle actin (α-SMA), fibroblast surface protein as well as fibroblastic markers. Carcinoma-associated fibroblasts (CAFs) are activated fibroblasts, biologically different from fibroblasts present in benign microenvironments in several important aspects [17,18]. CAFs are not passive bystanders in tumorigenesis and metastasis, but contribute actively to these processes [19]. Our knowledge on the role of resting and activated fibroblasts in cancer is still evolving. Here, we focus on the interaction between cancer cells and their microenvironments to study how the fibroblasts isolated from human gastric carcinomas facilitate tumorigenesis.

## Results

### The expression of TAGLN is upregulated in gastric carcinoma-associated fibroblasts

**Figure 1:**
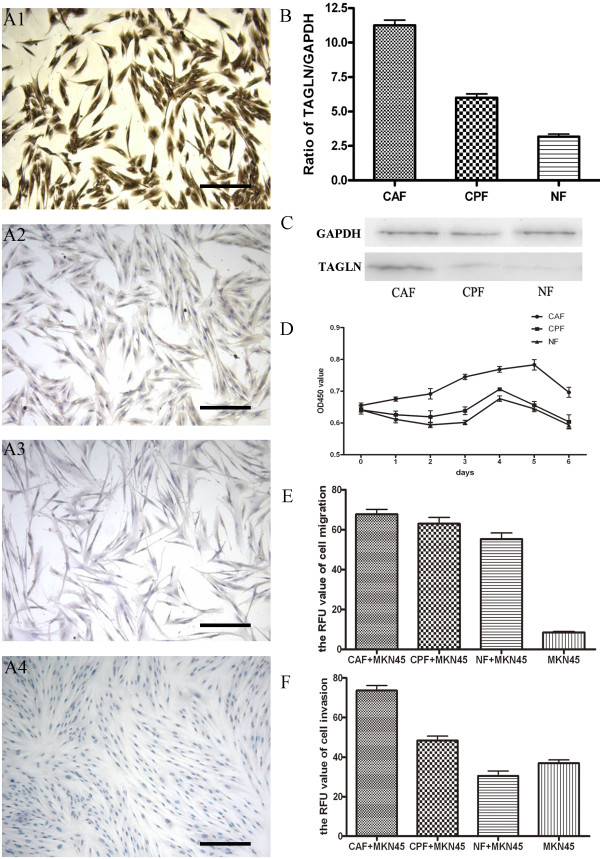
**The expression of TAGLN is upregulated in gastric cancer-associated fibroblasts. A**: characterization of gastric fibroblasts (ICC). a1: vimentin, positive. a2: α-SMA, positive. a3: fibronectin, positive. a4: cytokeratin, negative. Original magnifications: ×200. Scale bars, 100 μm. **B**: QRT-PCR analysis of TAGLN in CAF, CPF and NF. *, *P* < 0.05, **, *P* < 0.01, compared to NF. **C**: Western blot analysis of TAGLN in CAF, CPF and NF, TAGLN expression level was decreased successively. **D**: fibroblasts growth curve were determined by CCK-8 assay, CAFs grew quickier than the others. **E**: CAF had higher ability to enhance MKN-45’s migration ability than CPF and NF, ***, *P* < 0.001 compared to MKN-45. **F**: CAF had higher ability to enhance MKN-45’s invasion ability than CPF and NF, *, *P* < 0.05, **, *P* < 0.01, compared to MKN-45.

The carcinoma-associated fibroblasts (CAFs), counterpart fibroblasts (CPFs) and normal fibroblasts (NFs) were obtained from primary tissue culture using tissues from cancer-associated regions, non-cancer-associated stroma and normal mucosa, respectively. We then verified the purity of the various fibroblast populations by immunostaining. These fibroblast populations expressed high levls of fibroblastic markers such as vimentin, α-SMA and fibronectin. On the other hand, these cells did not express epithelial markers such as cytokeratin (Figure 1A). These data confirmed the purity of the fiborblasts. These observations indicate that the culture cells were predominantly fibroblasts were prepared with minimal contamination by other cells.

The mRNA and protein levels of TAGLN were lower in CPFs and NFs compared with CAFs (Figure 1B and C). We first showed that CAFs grew significantly more than CPFs and NFs in vitro cell proliferation (Figure 1D). Next, we carried out cell invasion and migration assay to test whether the invasion and migration ability of gastric cancer cells would be enhanced by fibroblasts. The experiment was designed to investigate the effects of stroma TAGLN, which was secreted by fibroblasts, on human gastric cancer cells. The internal levels of TAGLN in gastric cancer cells might interfere the experimental process. Hence, we choosed MKN-45 cell line, because its TAGLN expression level was the lowest in seven gastric cancer cell lines (SNU-1, AGS, NCI-N87, KATOIII, MKN-45, MKN-28 and SGC-7901; data not shown). Interestingly, the invasion and migration ability of MKN-45 increased in all the three groups (CAFs, CPFs and NFs), and increased most in the group with CAF (Figure 1E and F). The acquisition of migration and invasive behavior is one of steps in the metastatic process.

### CAFs promote tumor metastasis through upregulating TAGLN

**Figure 2:**
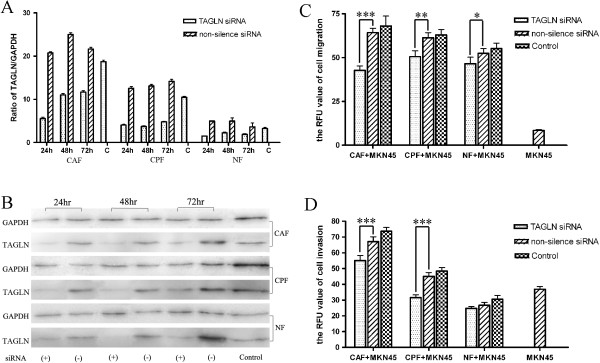
**CAFs enhance tumor cell line MKN-45 migraton and invasion through upregulated TAGLN. A**: TAGLN expression by QRT-PCR analysis after siRNA knockdown in fibroblasts. **B**: TAGLN expression by Western blot analysis after siRNA knockdown in fibroblasts. **C**: cell migration assay in vitro (fibroblasts with or without TAGLN siRNA interference). **D**: cell invasion assay in vitro (fibroblasts with or without TAGLN siRNA interference). Values represent the mean ± SD from at least three separate experiments, each conducted in triplicate. *, *P* < 0.05, **, *P* < 0.01, ***, *P* < 0.001, by one-way analysis of variance (ANOVA).

To examine the effect of TAGLN in fibrobalsts, we used RNAi to knock down TAGLN expression. CAFs, CPFs and NFs were transfected with TAGLN-specific or non-silence siRNA. RNA and protein were obtained at 24, 48 or 72 hrs after transfection, TAGLN mRNA and protein levels were investigated by QRT-PCR and Western blot. TAGLN mRNA and protein were significantly inhibited in cells treated with TAGLN-specific siRNA (Figure 2A and B).

We next investigated whether the invasion and migration ability of gastric cancer cells will be enhanced by the expression of TAGLN in fibroblasts, we carried out cell invasion and migration assay in vitro. The fibroblasts and MKN-45 were non-contactedly co-cultured. Interestingly, the invasion and migration ability of MKN-45 decreased immediately in the group with CAFs and decreased slightly in the group with CPFs and NFs. The results showed that the invasion and migration ability of MKN-45 can decrease through down-regulating the expression of TAGLN (Figure 2C and D).

**Figure 3:**
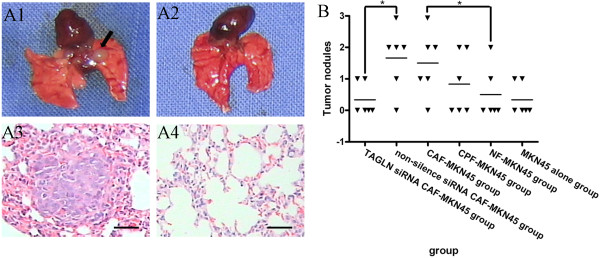
**TAGLN enhances tumor metastasis through human tumor xenograft model in vivo. A**: nude mice metastatic nodules in the lung. a1: lung metastatic nodules from tomur-bearing mouse. Arrow: metastatic nodule. a2: no metastatic nodule in lung. a3: H&E staining for lung metastatic nodule under bright-field microscope. Scale bars, 200 μm. a4: H&E staining for lung without metastatic nodule under bright-field microscope. Scale bars, 200 μm. **B**: quantification of nude mice lung metastatic nodules in various groups through tail intravenous injection. *, *P* < 0.05, by one-way analysis of variance (ANOVA).

In order to assess the contribution of CAFs-TAGLN to tumor metastasis in vivo, we emplyed a xenograft model. We mixed TAGLN siRNA CAFs, non-silenced CAFs, CPFs and NFs with MKN-45 human gastric cancer cells in a 2:1 ratio or MKN-45 alone and inoculated these cells through tail intravenous injection in immunodeficient nude mice. After 6 weeks, MKN-45 mixed with CAFs generated more lung metastatic nodules than MKN-45 mixed with TAGLN siRNA CAFs, CPFs and NFs, similar to MKN-45 mixed with non-silence siRNA CAFs (Table 1). These data suggested that CAFs show an increased ability to stimulate tumor metastasis (Figure 3A and B). All the observations indicated that in the presence of CAFs, tumors became more aggressive, suggesting that CAFs might play roles in promoting metastasis in gastric cancer.

**Table 1:** Nude mice lung metastatic nodules in various groups through tail intravenous injection (Each group contained 6 mice)

**groups**	**TAGLN siRNA CAF-MKN45**	**non-silence siRNA CAF-MKN45**	**CAF-MKN45**	**CPF-MKN45**	**NF-MKN45**	**MKN45 alone**
Number of tumor-bearing mice	2	5	5	3	2	2
Number of metastatic nodules	2	10	9	5	3	2

### TAGLN promotes tumor metastasis by upregulating MMP-2

**Figure 4:**
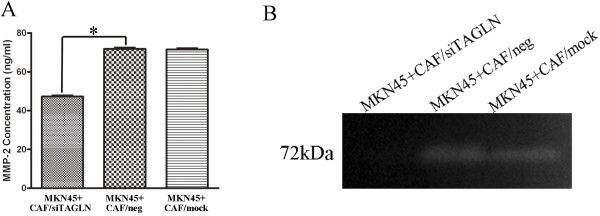
**TAGLN enhances tumor metastasis by upregulated MMP-2. A**: MMP-2 expression in CAF decresed obviously after TAGLN RNAi by ELISA. *, *P* < 0.05 by one-way analysis of variance (ANOVA). Error bars depict the standard error around the mean. **B**: knockdown TAGLN impaired the ability of CAF to secret MMP-2 by gelatin zymography.

Many studies have focused on the role of matrix metalloproteinases (MMPs) to the invasion of surrounding connective tissue and metastasis of tumor cells from the primary lesion to distant sites in tumor progression [20,21]. In our previous work, we examined MMP-1, MMP-2, MMP-3, MMP-7 and MMP-9 levels after TAGLN knock-down by siRNA through ELISA, only MMP-2 level was significantly down-regulated, other MMPs levels showed no change. Hence, we focued on MMP-2 in this study. As Figure 4A showed, compared to MKN-45 co-coultured with CAF/neg and CAF/mock, MMP-2 levels in supernatant from MKN-45 co-cultured with CAF/siTAGLN were significantly suppressed. We next examined the activity of MMP-2 using gelatin zymography assay. We found MMP-2 activity was dramatically inhibited in supernatant from MKN-45 co-cultured with CAF/siTAGLN than with CAF/neg and CAF/mock (Figure 4B). Thus, TAGLN may enhance tumor metastasis through the MMP-2 enzymes degrade the basement membranes (eg. collagen IV).

### TAGLN expression in human gastric cancer and its association with differentiation in gastric cancer patients

**Figure 5:**
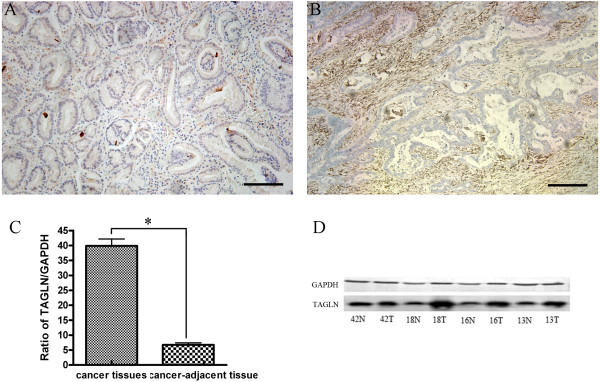
**Expression of TAGLN in human gastric cancer tissues. A**: TAGLN expression level in normal gastric tissue by IHC. Scale bars, 100 μm. **B**: TAGLN is overexpressed in the stroma of human gastric cancer by IHC. Scale bars, 100 μm. **C**: TAGLN mRNA is upregulated in human gastric cancer analyzed by QRT-PCR. **D**: TAGLN protein is upregulated in human gastric cancer analyzed by Western blot. Values represent the mean ± SD from at least three separate experiments, each conducted in triplicate. *, *P* < 0.001 by paried-samples *T* test.

To evaluate the TAGLN expression in gastric cancer, TAGLN immunostaining in 98 primary gastric cancer tissues were examined. TAGLN expression was upregulated in human gastric cancer stroma compared to normal gastric tissue (Figure 5A: normal gastric tissue; Figure 5B: gastric cancer tissue). The correlation of TAGLN expression with the clinicopathological features was shown in Table 2. TAGLN levels were higher in undifferentiated tumors (poorly differentiated adenocarcinomas, signet ring cell carcinomas and mucinous carcinomas) than that in differentiated ones (well and moderate adnocarcinomas) (*P* = 0.003). Moreover, TAGLN levels was correlated with lymph node metastasis (*P* = 0.029). There were no significant differences in tumor TAGLN expression according to the other clinicopathological features such as gender, age, tumor size, Lauren classification, T stage, distance metastasis and TNM stage (*P* ≥ 0.05).

**Table 2:** Clinicopathological associations of TAGLN expression in gastric cancer

	**Number of cases**	**TAGLN immunostaining**		**P**
		**Weak(+)**	**Strong(++)**	
Gender				0.778
Male	60	27	33	
Female	38	16	22	
Age				0.739
≤60y	46	21	25	
>60y	52	22	30	
Tumor size				0.852
≤5 cm	58	25	33	
>5 cm	40	18	22	
Differentiation				0.003
Well + Moderate	46	13	33	
Poor	52	30	22	
Lauren classification				0.059
Intestinal	40	13	27	
Diffuse	58	30	28	
T stage				0.142
T1 + T2	56	21	35	
T3 + T4	42	22	20	
Lymph node metastasis				0.029
N0	27	11	16	
N1	32	15	17	
N2	26	7	19	
N3	13	10	3	
Distant metastasis				0.709
Without	95	42	53	
With	3	1	2	
TNM stage				0.722
I	20	7	13	
II	25	11	14	
III	30	10	20	
IV	23	15	8	

Also, we used QRT-PCR and Western blot to evaluate the expression of TAGLN in gastric cancer tissues (Figure 5C and D). These results suggested that the TAGLN expression in gastric cancer tissues was much higher in gastric cancer than that in paired normal tissues.

## Discussion

Accumulating evidence demonstrates that cancer initiation and progression involve interactions between both tumor and stromal cells. Tumor cells can actively recruit stromal cells, such as vascular cells, smooth muscle cells and fibroblasts, into the tumor, thus generating microenvironment to foster tumor growth [16,22,23]. Fibroblasts often constitute the majority of the stromal cells within a gastric carcinoma, yet the functional contributions of these cells to tumorigenesis and tumor metastasis remain poorly understood.

TAGLN is one of the earliest markers of smooth muscle differentiation during embryogenesis [24], and previous studies related to TAGLN mainly focused on smooth muscle cells [25]. Pathological conditions accompanied with tissue remodeling and fibrogenesis, such as wound healing and neoplastic lesions, are characterized by the appearance of stromal cells with ultrastructural features intermediate between those of typical fibroblasts and those of smooth muscle cells. These fibroblasts exhibit phenotypic and functional features of smooth muscle cells and hence are named myofibroblasts [26], such as CAFs. In Our study, TAGLN overexpression was also observed in CAFs. It is reported that the TAGLN protein is overexpressed in gastric cancer when protein expression spectrum of gastric cancer was analyzed using two-dimensional gel electrophoresis (2-DE) [27]. Furthermore, the TAGLN protein is one of the two tumor-associated antigens which were identified in serum of kidney carcinoma patients by Klade using 2-DE [28]. These reports are consistent with the findings that we have observed using real-time PCR and Western Blot. Interestingly, we found that TAGLN was mainly detected in fibroblasts derived from tumor stroma, rather than in tumor cells. Similarly, other investigators discovered that the TAGLN is not expressed in the malignant cells but in mesenchymal cells of the tumor stroma [27].

Stromal fibroblasts regulate endothelial or epithelial cell behavior through direct and indirect cell-cel1 interactions. Activation of host stroma1 microenvironment is thought to be a critical step in tumor growth and progression [13,29]. Proteins secreted by stromal cell in the tumors may positively or negatively affect tumor progression. The most prominent actions of TAGLN may favor tumor cell motility and invasion [7,30,31]. We found that the TAGLN was significantly increased in lymph node metastasis group than lymph node negative group. To c1arify the role of TAGLN in stromal fibroblasts during gastric cancer tumorigenesis and metastasis, we silenced the TAGLN expression in human CAFs. It was indicated that only the MKN-45 co-cultured with CAFs expressing high level TAGLN exhibited much higher invasion and migration ability. Fibroblasts which TAGLN had been knocked down did not display this function. Taken together, we suggest that TAGLN might be responsible for the enhancement of gastric cancer cell metastasis via stromal cells such as CAFs.

TAGLN expression is increased significantly in gastric CAFs. CAFs with increased TAGLN expression levels may enhance tumor cell invasion and migration ability which promote an enhanced expression of MMP-2. The stimulation of MMP expression in fibroblasts either after direct contact with tumor cells or after contact with tumor cell-derived soluble factors from tumors originating from epithelial cells has been shown in several studies [32-35]. Clarifying the underlying mechanisms may therefore attenuate the tumor recurrence and metastasis in gastric cancer patients.

## Conclusions

In summary, our study demonstrates that CAFs may promote gastric cancer cell migration and invasion via increased MMP-2 production caused by TAGLN upregulation.

## Methods

### Tissue samples

A total of 40 pairs of cancer tissues and corresponding normal mucosa for QRT-PCR and Western blot and 98 primary gastric cancers for immunostaining were collected from surgically removed gastric cancer diagnosed at the Department of Surgery, Ruijin hospital, Shanghai Jiao Tong University School of Medicine. Normal mucosa samples were taken from the margin of the resection where the tumors located at least over 6 cm apart from it. This study protocol was approved by the independent ethics committee of Ruijin Hospital, Shanghai Jiaotong University, School of Medicine. The informed consent was written. All samples were snap frozen in liquid nitrogen overnight and kept at −80°C before use. 4% Formaldehyde-fixed tissue sections of both tumor and mucosa were stained with H&E and examined and verified histopathologically by two pathologists. Each specimen was attributed a diagnosis and scored for Lauren classification, differentiation, pTNM stage. Tumor size, location and other clinical pathological characteristics were obtained from clinical records with patient permission.

### Cell lines

Gastric cancer cell lines SNU-1 (ATCC: CRL-5971), AGS (ATCC: CRL-1739), NCI-N87 (ATCC: CRL-5822) and KATOIII (ATCC: HTB-103) were obtained from the American Type Culture Collection (Manassas, VA, USA). Another 3 gastric cancer cell lines, MKN-45, MKN-28 and SGC-7901, were preserved at our institute. All cell lines were maintained in RPMI-1640 supplemented with 10% fetal bovine serum (FBS).

### Antibodies

Primary antibodies used for staining included human specific anti-vimentin (Abcam, Cambridge, UK), anti-fibronectin (Abcam, Cambridge, UK), anti-α-smooth-muscle actin (Sigma Chemical, St Louis, MO, USA), anti-pan-cytokeratin (Abcam, Cambridge, UK), anti-SM22 (Abcam, Cambridge, UK), and mouse anti-GAPDH (Kangchen, China).

### Isolation of human gastric fibroblasts

Fibroblasts were isolated from cancer- and non-cancer-associated regions of gastric tissues dissected from patients with gastric cancer during radical gastric resection from the Department of Surgery, Ruijin hospital, Shanghai Jiao Tong University School of Medcine. The cancer-associated regions were selected to be minimally necrotic regions of the tumor mass. Non-cancer-associated stroma, which was isolated from tissue at least 2 cm distal to the outer margin of the cancer mass. Normal mucosa samples were taken from the margin of the resection where the tumors located at least over 6 cm apart from it. Tissues were minced into organoids of approximately 1 mm^3^ and seeded on uncoated plastic material in RPMI 1640 medium containing human basic fibroblast growth factor, 20% fetal calf serum (FCS) and penicillin and streptomycin as antibiotics. These conditions produced a homogenous fibroblastic cell population after 7 days of culture. Each fibroblast was then expanded at a 1:3 ratio by trypsin-EDTA into 10 cm petri dishes. We used fibroblasts passaged for up to 8 population doublings (PDs) for subsequent experiments, in order to minimize clonal selection and culture stress which could occur during extended tissue culture.

### Immunohistochemical staining (IHC)

Cells or tissues were fixed for 30 min with 4% formalin and rinsed with phosphate buffered saline (PBS). After endogenous peroxidase activity was quenched with 0.3% H_2_O_2_, cells were blocked with normal nonimmune serum for 30 min prior to being incubated with primary antibody at 37°C for 2 hrs. Cells or tissues were then labeled with streptavidin-biotin-horseradish peroxidase staining kit (Dako, Carpinteria, CA, USA). The presence of labeled peroxidase on the sections was visualized by incubation with 3,3^′^-diaminobenzidine, DAB + substrate chromogen and the sections were counterstained with hematoxylin. Negative control slides were performed by omission of the primary antibody.

TAGLN expression was assessed by the intensity of stained cells, and determined in two categories (weak positive and strong positive). The staining intensity was classified according to four grades (intensity scores): no staining (grade 0), light brown staining (grade 1), brown staining (grade 2), and dark brown staining (grade 3). Grade 0 and grade 1 was defined as weak positive, grade 2 and grade 3 were defined as strong positive.

### Quantitative real-time PCR (QRT-PCR)

Total RNA was extracted using Trizol reagent (Invitrogen, Carlsbad, CA, USA) following the manufacturer’s instructions. cDNA was obtained with 1 μg RNA using Reverse Transcription System Kit (Promega, Madison, WI, USA) and QRT-PCR was performed by the SYBR Green PCR core Reagent kit (Applied Biosystems, Warrington, UK). Primers specific to TAGLN were as follows: 5^′^-GAGCAAGCTGGTGAACAGCC-3^′^ (upper), 5^′^-GACCATGGAGGGTGGGT TCT-3^′^ (lower). Glyceral-dehyde-3-phosphate dehydrogenase (GAPDH) was used as the endogenous reference. Its sequences were 5^′^-GGACCTGACCTGCCGTCT AG-3^′^ (upper), 5^′^-GTAGCCCAGGATGCCCTTGA-3^′^ (lower). QRT-PCR for quantitation of the mRNA levels of TAGLN was done on an ABI Prism 7000 (Applied Biosystems, Foster City, CA, USA). Data were analyzed by using the comparative Ct method. Specificity of resulting PCR products was confirmed by melting curves.

### Western blot

Cells were harvested and lysed with mammalian protein extraction reagent (Pierce, Rockford, IL, USA). Protein concentrations were determined with a bicinchoninic acid (BCA) protein assay kit (Pierce, Rockford, IL, USA). Samples containing 50 μg of total protein were loaded onto each lane of 12% acrylamide gel in a minigel apparatus (Bio-Rad, Richmond, CA, USA). The separated proteins were transferred to a polyvinylidene difluoride (PVDF) membrane (Bio-Rad, Richmond, CA, USA). After being incubated with primary antibody (1:1,000) and HRP-conjugated secondary antibody (1:5,000) respectively, immune complexes were detected by the enhanced chemiluminescent (ECL) system (Millipore, Bedford, MA, USA).

### Enzyme-linked immunosorbent assay (ELISA)

The protein levels of MMP-2 in supernatants were measured by an ELISA kit (R&D Systems Inc., Minneapolis, MN, USA) according to the manufacturer’s instructions.

### Cell growth assay

The different groups of cells (1 × 10^3^) were seeded into 96-well plates in triplicate. The number of viable cells was determined daily using the Cell Counting Kit (CCK-8) by utilizing a highly water-soluble tetrazolium salt WST-8 [2-(2-methoxy-4- nitrophenyl)-3-(4-nitrophenyl)-5-(2,4-disulfophenyl)-2H-tetrazolium, monosodium salt] (Dojindo, Japan). Briefly, 20 μl of CCK-8 solution was added into plates, absorbance at 450 nm was measured after 4 hrs incubation.

### Assessment of tumor invasion and migration

Tumor invasion and migration assay were performed with QCMTM24-well invasion kit (Millipore, Bedford, MA, USA) and QCMTM24-well migration kit (Millipore, Bedford, MA, USA) according to the protocol provided by the manufacturer, respectively. Add prepared MKN-45 cell suspension (2 × 10^5^ cells/well) to the upper chamber and add pre-suspended fibroblasts (5 × 10^4^ cells/well in RPMI 1640 medium containing 20% FBS) to the lower chamber. The TAGLN of some fibroblasts in lower chambers was inhibited using siRNA 24 hrs later. MKN-45 and fibroblasts were co-cultured non-contactedly for 48 hrs, then lysised the cells and determined RFU values with a fluorescence plate reader using 480/520 nm filter set.

### TAGLN siRNA

The Qiagen software was used to design RNAi sequences targeting human TAGLN (Accession no. NM_003186), and the siRNA sequence with the highest putative efficacy (sense: 5^′^-AAAUCGAGAAGAAGUAUGAdtdt-3^′^, antisense: 5^′^-UCAUAC UUCUUCUCGAUUUdtdt-3^′^) was synthesized by Shanghai GeneChem Co, Ltd. siRNA with randomized sequence (sense: 5^′^-UUCUCCGAACGUGUCACG Utt-3^′^, antisense: 5^′^-ACGUGACACGUUCGGAGAAtt-3^′^) against no gene (scrambled siRNA group) was transfected as internal control. For experiments, cells were transfected with the DOTAP liposomal transfection reagent (Roche, Germany). Cells were seeded at 2 × 10^5^ cells/well in 6-well plates. After 24 hrs when cells were in the phase of log growth, 250 μl Opti-MEM I was mixed with 5 μl of 20 μM siRNA duplex, while another 250 μl Opti-MEM I was separately incubated with 11.88 μl of DOTAP liposome. The two mixtures were gently mixed, and incubated for about 30 min at room temperature. For transfection, the entire mixture was added to each well in 1.5 ml of fresh medium without antibiotics. The final transfected concentration of siRNA was 20 nM. Cells were collected for further assay at 24, 48 and 72 hrs after transfection.

### Gelatin zymography

The culture supernatants were harvested at 24 hrs and mixed with a gel sample buffer (0.5 M Tris–HCl, glycerol, 10% sodium dodecyl sulfate [SDS], β-mercaptoethanol, and 0.5% bromophenol blue). Ten micrograms of protein were taken by SDS-PAGE separation; the SDS-PAGE gels contained 0.1% gelatin (Sigma Chemical, St Louis, MO, USA). After electrophoresis, the gels were washed in 50 mM Tris buffer containing 2.5% Triton X-100. The gels were incubated for an additional 24 hrs in incubation fluid (50 mM Tris buffer [pH 7.6], 10 mM CaCl_2_, and 200 mM NaCl). The gels were stained with 0.5% Coomassie blue, white bands (72 kDa) on a blue background indicated zones of digestion corresponding to the presence of MMP-2.

### Mouse model

Female BALB/c *nu/nu* nude mice, age-matched between 4–5 weeks (Institute of Zoology Chinese Academy of Sciences), were housed at a specific pathogen-free environment in the Animal Laboratory Unit, Shanghai Jiao Tong University School of Medicine, China. Fibroblasts and MKN-45 were mixed at the ratio of 1:4 within 0.1 ml PBS and injected into the lateral tail vein. Six mice were included in each group in all experiments, and each experiment was performed twice. Animals were sacrificed and lung metastatic nodules were recorded 6 weeks after tumor cell implantation. Tumor specimens were collected, mixed in formalin, embedded in paraffin, and subjected to H&E staining. Mice were manipulated and cared according to the guidelines and protocols approved by the Medical Experimental Animal Care Commission of Shanghai Jiaotong University School of Medicine.

### Statistical analysis

All experiments were repeated at least three times. Results were summarized as means ± standard deviation (SD). The correlation between TAGLN expression and clinicopathological parameters was calculated with two-sided chi-square test. *P* < 0.05 was selected as the statistically signignificant value. SPSS version 11.0 software was used for all analyses.

## Competing interests

The authors declare no competing interest in this paper.

## Authors’ contributions

BQY and XHC conducted the experiments. BQY and BYL made the hypothesis, designed the experiments and wrote the manuscript. JFL, YQ and LPS provided assistance in some of the experiments. BQY and YBP collect the specimens. JH, JY, YYY, QLG and ZGZ supervised the study. All authors read and approved the final manuscript.
